# Current methods and future needs for visible and non-visible detection of plant stress responses

**DOI:** 10.3389/fpls.2025.1585413

**Published:** 2025-09-29

**Authors:** Julian Cooper, Kevin Propst, Cory D. Hirsch

**Affiliations:** Department of Plant Pathology, University of Minnesota, Saint Paul, MN, United States

**Keywords:** stress detection, multi-omics, remote sensing, machine learning, phenotyping

## Abstract

As climate change alters the frequency, intensity, and co-occurrences of abiotic and biotic stresses, the effective and efficient detection of plant stress responses and resistance mechanisms is critical for safeguarding global food security. Stressful environments elicit both visible and non-visible changes in plants. Cellular and subcellular changes, often invisible to the naked eye, can serve as indicators of stress and can be quantified using molecular, ionomic, metabolomic, genomic, and transcriptomic methods. In contrast, visible responses such as discoloration, morphological changes, and disease symptoms can be monitored efficiently through atmospheric, aerial, and terrestrial remote sensing platforms. Phenotyping at the whole-plant and organ levels offers valuable insights for diagnosing stress *in situ*, providing opportunities to study plant resistance and acclimation strategies under realistic conditions. However, the complexity of plant stress responses, spanning microscopic to macroscopic scales and diverse biological processes, make it challenging for any single technology to comprehensively capture the full spectrum of reactions. Furthermore, the rising prevalence of multifactorial stress conditions highlights the need for research on synergistic and antagonistic interactions between stress factors. To effectively mitigate the impacts of stress on agriculture, future research must prioritize integrative multi-omic approaches that connect cellular and subcellular processes with morphological and phenological stress responses.

## 1 Introduction

Anthropogenic climate change is projected to negatively affect the availability and productivity of agricultural land in the coming decades (Gowda et al., 2018). Rising atmospheric carbon dioxide levels have been linked to an increase in the frequency and intensity of extreme weather events and temperature fluctuations (Amirkhani et al., 2022). Additionally, shifting environmental conditions have expanded the habitable ranges of many phytopathogens and contributed to the emergence of novel plant diseases (Nnadi and Carter, 2021). Climate disturbances also increase the likelihood of concurrent stressors, such as heat and drought or flooding and disease (Rillig et al., 2021). The interplay of abiotic and biotic stressors is already affecting food production, availability, and nutritional quality, with these impacts expected to intensify in the future (Brown et al., 2015).

To effectively manage these challenges, robust systems for stress detection and characterization are needed. Stress responses in plants manifest across multiple scales, ranging from non-visible cellular and subcellular changes to visible symptoms at the organ, plant, plot, and field levels. Currently, a variety of stress detection methods are available, each with specific strengths and limitations. However, existing approaches may fall short in detecting rapidly emerging stressors, concurrent stress events, or plant resistance mechanisms that could be used for crop improvement. Addressing these gaps is essential to mitigate the impacts of climate change on global food security.

In this review, we assess the current methodologies used to detect and characterize plant responses to abiotic and biotic stresses. We focus on molecular, ionomic, metabolomic, transcriptomic, and genomic approaches for analyzing non-visible stress responses, as well as remote sensing technologies and image analysis techniques for monitoring visible symptoms. Finally, we highlight the need for innovative experimental designs and integrative technologies to generate and utilize stress response data, enabling more comprehensive strategies to address future agricultural challenges.

## 2 Detection of non-visible stress factors and plant response mechanisms

**Figure 1:**
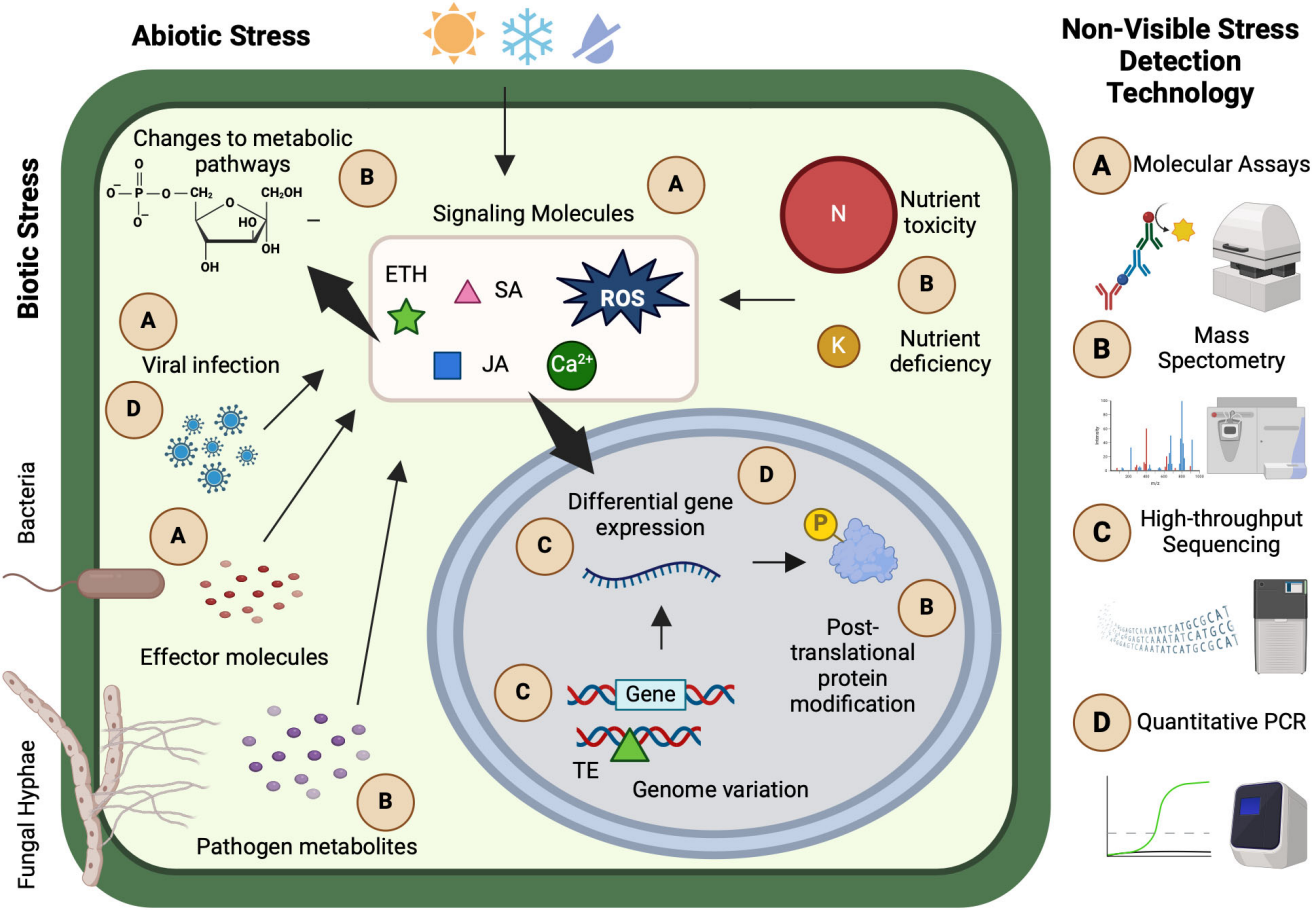
Selected examples of non-visible stress plant responses, detection technologies, and applications. **(A)** Luminescence and enzyme immunological molecular assays can be used to detect plant pathogens and host and pathogen signaling molecules. **(B)** Mass spectrometry has various applications in ionomics, metabolomics, and proteomics such as detecting nutrient toxicity or deficiency, pathogen and host metabolites, and post-translational protein modifications. **(C)** High-throughput sequencing technologies facilitate rapid study of genomic variation and differential gene expression that coincide with plant stress responses. **(D)** Approaches such as quantitative polymerase chain reactions are useful for the targeted study of specific sequences, such as genes directly involved in stress response or viral infections. Many non-visible stress detection technologies are primarily conducted in controlled environments or laboratory settings. This figure was created with www.biorender.com.

Abiotic and biotic stresses trigger a cascade of internal reactions in plants, including molecular signaling, hormonal responses, and differential gene expression (**Figure 1**). Following stress exposure, plants typically undergo three fundamental stages of response. In the alarm phase, a wide range of cellular and sub-cellular processes are activated, leading to initial changes in host gene expression. During the acclimation phase, these gene expression changes drive the production of stress-responsive proteins and metabolites. Finally, in the resistance phase, the plant’s stress phenotype is fully established, reflecting its capacity to adapt or resist the adverse conditions (Vítámvás et al., 2015).

These non-visible processes provide opportunities to identify plants undergoing different stages of the stress response and to characterize mechanisms of plant resistance. However, studying these intricate pathways presents challenges due to their small scale and complexity. To investigate the myriad of molecular, ionomic, metabolomic, proteomic, genomic, and transcriptomic reactions associated with plant stress, researchers have developed and deployed a wide range of detection technologies and protocols. These tools enable precise characterization of stress responses at the cellular and subcellular levels, offering insights into the mechanisms plants use to sense, respond to, and mitigate environmental challenges.

### 2.1 Biological assays for detection of stress response related molecules

Biological assays are widely used to quantify specific molecules or compounds and evaluate their effects on living tissue (**Figure 1A**). In plants, exposure to biotic or abiotic stress triggers molecular changes, including fluctuations in intracellular Ca^2+^ concentrations and reactive oxygen species (ROS) production, which initiate the alarm phase of the stress response and regulate stress-responsive gene expression (Vítámvás et al., 2015; Verma et al., 2016). The timing and magnitude of Ca^2+^ and ROS production vary depending on the stressor, but their rapid production following exposure necessitates specialized detection techniques (Smith and Heese, 2014; Riveras et al., 2015).

Chemiluminescence-based bioassays provide a simple and effective approach for quantifying Ca^2+^ and ROS levels by measuring light emission from targeted chemical reactions. These assays have been used to monitor intracellular Ca^2+^ changes in response to stressors such as nitrate and heat (Zheng et al., 2012; Riveras et al., 2015). Variations of luminescence-based assays also enable pathogen detection. For example, a luminometer-based ROS bioassay was developed to quantify dose-dependent ROS production in Arabidopsis leaf tissue following *Pseudomonas syringae* infection, advancing research on host-pathogen interactions and resistance mechanisms (Smith and Heese, 2014). However, because chemiluminescence assays require destructive sampling, their application is limited for time-series studies unless multiple samples are collected.

Fluorescence-based bioassays, in contrast, utilize high-energy photon absorption and subsequent low-energy emission to track plant responses non-destructively. Chlorophyll fluorescence imaging has been widely applied to assess abiotic stress impacts, including nutrient deficiency, heat stress, and drought (Zivcak et al., 2013; Bayçu et al., 2018; Jat et al., 2024). Chlorophyll fluorescence is based on the principle that a portion of absorbed light energy in photosystem II (PSII) is reemitted as fluorescence. Under stress, alterations in PSII efficiency can be quantified using the Fv/Fm ratio, which reflects the maximum quantum yield of PSII photochemistry. Declines in Fv/Fm are indicative of stress-induced photoinhibition and are often correlated with oxidative stress, nutrient imbalances, or water deficiency. This non-destructive method is commonly used in parallel with biochemical assays such as antioxidant enzyme activity or metabolite quantification to validate physiological stress responses. When coupled with proximal sensors, chlorophyll fluorescence imaging enables spatial and temporal monitoring of plant stress under both controlled and field conditions (Arief et al., 2023). These methods also facilitate real-time monitoring of biotic stress, particularly when pathogens are genetically engineered to express fluorescent proteins. For instance, *Phytophthora capsici* strains expressing red fluorescence protein enabled *in vivo* tracking of pathogen growth and host resistance evaluation in cucumber and pepper plants (Zhang et al., 2021).

Non-luminescence-based assays, such as enzyme-linked immunosorbent assays (ELISA), are powerful tools for detecting plant stress markers. ELISA uses antigen-antibody interactions to detect and quantify pathogens, making it a common method for studying plant viral infections. Immunoassays can detect pathogens at very low concentrations, but their application is limited to known pathogens for which antibodies are available (Clark, 1981). Beyond pathogen detection, enzyme immunoassays have been used to quantify stress-related hormones based on specific antibody-substrate interactions (Pradko et al., 2015). Notably, ELISA has been used to measure heat shock proteins, which play a crucial role in activating multimodal stress-response gene expression (Wang et al., 2015; Olate et al., 2018). While molecular bioassays provide precise detection of stress signals and byproducts, their high specificity often limits broad applicability. Integrating multiple bioassays with complementary detection methods can enhance our ability to characterize plant stress responses comprehensively.

### 2.2 Characterization and response of the plant ionome, metabolome, and proteome during stress exposure

Rather than targeting specific stress signals through bioassays, large-scale omic approaches enable the comprehensive characterization of molecular and biochemical stress profiles in plants (van Dijk et al., 2021). In this review, we focus on ionomics, metabolomics, and proteomics, which provide valuable insights into plant responses to abiotic and biotic stressors. Ionomics is the study of an organism’s elemental composition and nutrient dynamics. Nutrient imbalances, whether deficiencies or toxicities, can serve as abiotic stressors, disrupting plant growth and physiological function. Macronutrients such as nitrogen, phosphorus, and potassium, as well as micronutrients like boron, zinc, and iron, play essential roles in plant health (Shrivastav et al., 2020). Additionally, nutrient deficiencies have been linked to increased susceptibility to biotic stressors, as weakened plants may be more vulnerable to pathogens and pests (Mur et al., 2017; Tripathi et al., 2022). Metabolomics focuses on small-molecule metabolites that function as intermediates and end products of cellular processes. Metabolomic profiling can reveal plant-produced metabolites that mediate stress responses, as well as toxic metabolites synthesized by pathogens and pests. Proteomics involves the large-scale study of proteins, encompassing their abundance, modifications, and interactions (Kosová et al., 2018). Protein compositions dynamically shift in response to stress, making proteomic profiling a crucial tool for understanding functional responses at different stress stages (Kosová et al., 2015).

Mass spectrometry (MS) is a powerful tool for ionomic, metabolomic, and proteomic analyses, enabling the simultaneous detection of multiple elements or molecules based on their mass-to-charge ratio (**Figure 1B**) (Sparkman, 2000). Various MS techniques are tailored to different applications. Gas chromatography-MS (GC-MS) is commonly used in ionomics. This technique vaporizes and separates molecules via gas chromatography before ionizing them for MS detection. However, the high temperatures involved can degrade certain metabolites and proteins, making GC-MS less suitable for metabolomics or proteomics research (Fang et al., 2015). Instead, liquid chromatography-MS (LC-MS) is preferred for studying organic compounds. This method maintains molecular integrity by dissolving compounds in an organic solvent and water mixture before ionization (Zhou et al., 2012). Additional MS methods and analytical techniques are detailed in a review by Boughton et al., 2016.

Mass spectrometry has been applied in a variety of plant stress research. In ionomics, MS has been used to analyze elemental distributions in plants under stress, such as measuring nutrient levels in cotton seedlings exposed to salt stress (Guo et al., 2019). In metabolomics, MS has enabled research into stress-induced changes in metabolism and photosynthesis pathways (Kumari et al., 2015; Pang et al., 2016). Additionally, MS is a powerful tool not only for detecting stress-induced metabolic changes in host tissues, but also for identifying pathogen derived mycotoxins in infected grains, including following *Fusarium graminearum* infection in wheat (Wang et al., 2021). Proteomic MS-based studies have revealed post-translational modifications and tissue-specific protein responses to stress (Liu et al., 2019). In one example, proteomic profiling using LC-MS identified important nucleolar proteins for ribosome assembly that helped restore protein synthesis following heat stress (Muñoz-Díaz et al., 2024).

Despite its advantages, MS has limitations. Signal intensities can be affected by environmental conditions during testing, requiring careful data normalization to account for machine drift (Matsuda, 2016). Additionally, identifying unknown metabolites from raw MS data often requires further computational analysis, such as fragmentation modeling (Allen et al., 2016).

One alternative approach is nuclear magnetic resonance (NMR) spectroscopy. NMR quantifies molecular structures by measuring electromagnetic radiation emitted by atomic nuclei. This technique offers high-throughput, nondestructive analysis with minimal sample preparation (Kim et al., 2010) and has been used to characterize stress-related molecules and resistance pathways (Mirnezhad et al., 2010; Zhao et al., 2016). However, because NMR relies on detecting small energy differences in nuclear spin states, it requires relatively large sample amounts and is less sensitive than MS for identifying low-abundance metabolites (David and Rostkowski, 2020). As a result, there is often limited overlap between the molecules identified by MS and NMR. Using both methods in tandem has been shown to be the most effective strategy for capturing both the most abundant and most readily ionizable compounds in a sample (Bhinderwala et al., 2018).

Raman spectroscopy is another valuable tool for characterizing stress response, particularly when combined with other spectroscopic platforms such as NIR or hyperspectral imaging. This technique detects vibrational energy shifts in molecules, allowing for nondestructive identification of biochemical changes in plant tissues with minimal sample preparation. Raman has been applied to monitor nutrient deficiencies and to detect biotic stress, often at earlier stages than visible symptom onset (Egging et al., 2018; Huang et al., 2020). Recent advances in portable Raman spectrometers and surface-enhanced Raman spectroscopy have further expanded its utility for in field stress detection (Yeturu et al., 2016). Because Raman spectra capture complementary biochemical information relative to MS and NMR, integrating these approaches can provide a more complete understanding of stress-induced metabolic reprogramming and host-pathogen interactions.

Gel-based proteomic methods, such as two-dimensional differential gel electrophoresis (2D-DIGE) and western blotting, provide an alternative to MS-based proteomic analysis (Aslam et al., 2017; Abdallah et al., 2012; Dannfald et al., 2025). These techniques separate and quantify proteins based on charge and molecular weight, making them valuable for complex protein samples. The use of gel-based proteomic methods has led to breakthroughs in plant protein expression in response to abiotic and biotic stress. A study on drought-stressed barley crowns identified 105 differentially accumulated proteins associated with growth regulation, energy metabolism, and stress acclimation (Vítámvás et al., 2015). Similar proteomic studies have identified key proteins involved in plant responses to flooding, cold, salinity, and other abiotic stresses (Gharechahi et al., 2014; Yin et al., 2014; Kosová et al., 2018). Gel-based proteomics has also been instrumental in studying plant-pathogen interactions, identifying proteins involved in plant immune responses (Sidiq et al., 2022).

Each approach, ionomics, metabolomics, and proteomics, provides unique insights into plant stress responses. While MS remains a primary tool for high-resolution molecular characterization, complementary techniques like NMR and gel-based proteomics help overcome MS limitations. Integrating multiple strategies will be essential for advancing our understanding of plant stress physiology and developing more resilient crop varieties.

### 2.3 Identification of genomic and transcriptomic functional stress resistance mechanisms

Plants undergo extensive genomic and transcriptional changes in response to stress, providing insights into the molecular mechanisms underlying resistance and acclimation. Genomic and transcriptomic approaches help identify stress-responsive genes, genetic markers, and regulatory networks involved in plant defense (**Figure 1C**).

Genomics, the study of genome structure, function, and evolution (Muthamilarasan et al., 2019), has become an essential tool in plant stress research due to advancements in sequencing technologies. Linkage and association mapping are commonly used to detect quantitative trait loci and genetic markers associated with stress tolerance traits (Jamann et al., 2015). These analyses have facilitated the identification of genes or loci linked to disease resistance and heat and salinity tolerance (Cooper et al., 2019; Huang et al., 2018; Molero et al., 2023). Another important approach is resistance gene enrichment sequencing (RenSeq), which selectively targets genomic regions encoding proteins with conserved disease resistance domains, such as nucleotide-binding and leucine-rich repeat motifs. This technique is particularly useful for identifying presence-absence variation in resistance genes within populations, aiding in the discovery of novel resistance alleles (Jupe et al., 2013; Andolfo et al., 2014).

Beyond protein-coding genes, non-coding genomic regions also play a role in stress responses. Transposable elements (TEs) can influence gene expression by inserting into coding or regulatory regions (Hirsch and Springer 2017). TE mobilization can serve as an adaptive mechanism under stress conditions. For example, the activation of the *ONSEN* TE in Arabidopsis alters gene expression to enhance heat tolerance (Roquis et al., 2021). In addition, the epigenetic status of TEs can induce phenotypic plasticity and facilitate stress acclimation and tolerance (Negi et al., 2016).

Transcriptomics examines gene expression patterns by quantifying mRNA abundance. High-throughput sequencing enables genome-wide identification of differentially expressed genes associated with stress resilience. This approach has been used to identify genes responsible for resistance and susceptibility to pathogens and abiotic stressors, such as nutrient deficiencies and extreme temperatures (Razzaque et al., 2019; Buhrow et al., 2021). Comparative transcriptomics has been instrumental in identifying functional resistance mechanisms. For example, RNA-sequencing of drought-tolerance and drought-sensitive sorghum varieties revealed genes associated with water-use efficiency (Fracasso et al., 2016). Large-scale transcriptomic databases have further expanded the ability to compare stress-related gene expression across multiple plant species. One such example is a plant stress-specific transcriptome database that compiled RNA-sequencing data for 12 species across 133 unique stress and development conditions, providing an organized and accessible resource for stress research (Li et al., 2018). While this review focused on transcriptomics, whole genome assemblies, comparative genomics, and epigenetic studies also play critical roles in understanding plant stress responses.

In addition to large-scale genomic and transcriptomic studies, targeted approaches remain valuable for stress detection and functional analysis. Polymerase chain reaction (PCR) allows for the amplification and study of specific DNA sequences from stress-responsive genes (**Figure 1D**) (Bhat and Rao, 2020). Quantitative PCR (qPCR) has been widely used to detect biotic stress by amplifying pathogen DNA from host tissue. For example, qPCR assays have successfully quantified Aphanomyces infection in plant roots and soil samples (Botkin et al., 2022). This technique is also useful for measuring stress-induced gene expression, allowing researchers to identify putative resistance mechanisms through differential mRNA quantification (W. Xu et al., 2020).

Despite their advantages, genomic and transcriptomic methods have limitations. Many approaches require a high-quality reference genome, which is not available for all plant species (Kress et al., 2022). Additionally, the analysis of sequencing data relies on computationally intensive bioinformatic workflows, which often require specialized training and high-performance computing resources.

### 2.4 Challenges in microscopic stress detection

Despite the wide range of tools available for studying microscopic changes in plants under stress, several key challenges remain. One of the primary limitations is the reliance on destructive sampling, which restricts the ability to monitor dynamic stress responses non-invasively and continuously over time (Ang and Lew, 2022). Temporal variation in stress onset and duration can further complicate analyses, particularly transcriptomics, since gene expression patterns may shift during the time needed to sample large populations and experimental replicates. Spatial variability within experimental settings, driven by differences in macro- and micro-climate, soil composition, or rhizosphere interactions, can also introduce confounding effects, especially in field trials. Logistical hurdles, such as the need for dry ice or liquid nitrogen for sample preservation, add further complexity to field-based workflows and can limit accessibility in resource-constrained environments. In addition, methodological constraints associated with ionomic, metabolomic, and proteomic techniques, such as sample throughput, sensitivity to environmental noise, and the need for expensive instrumentation, can limit their utility for real-time or *in situ* detection of non-visible stress responses (Galieni et al., 2020). While microscopic approaches have been essential for uncovering molecular mechanisms of stress tolerance, their full potential will only be realized through integration with complementary techniques such as real-time phenotyping, portable diagnostic tools, and multi-omic data pipelines. This integration is crucial for bridging the gap between laboratory discoveries and field-scale applications, ultimately accelerating the development of stress-resilient crops.

## 3 Platforms and sensors for remote detection of stressors and phenotypes

Stress-induced changes at the cellular and molecular levels, including disruptions to hormonal pathways, gene expression, and metabolic processes, often lead to visible phenotypic alterations in plant growth and development. These macroscopic effects can serve as key indicators of plant health and stress responses. For instance, nutrient deficiencies can reduce chlorophyll content, accelerating leaf senescence and ultimately decreasing total biomass (Huang et al., 2020). Similarly, plant detection of biotic pathogens can trigger the production of ROS and activate resistance genes, leading to a hypersensitive response characterized by localized cell death and necrotic lesions (Balint-Kurti, 2019). The ability to detect stress-induced morphological and physiological variations is critical for scouting and mitigating abiotic and biotic stresses, as well as for identifying stress-resilient germplasm.

### 3.1 Scales of visible stress detection imaging platforms

**Figure 2:**
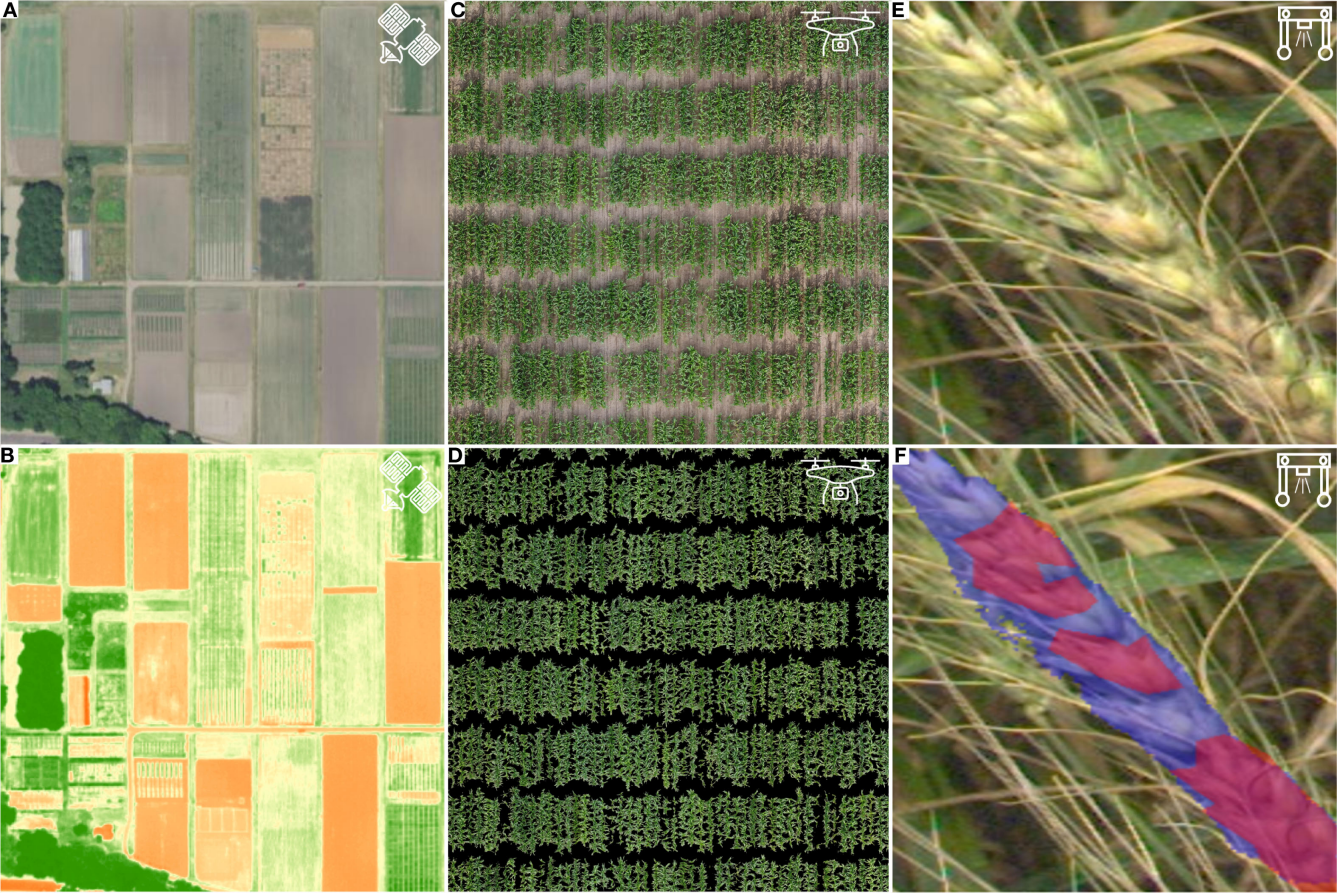
Example representation of different scales of stress detection and response targets. **(A)** Atmospheric satellite image of the University of Minnesota Saint Paul Agricultural Experiment Station acquired by the USA National Agriculture Image Program. **(B)** Atmospheric satellite image of the University of Minnesota Saint Paul Agricultural Experiment Station acquired by the USA National Agriculture Image Program overlaid with Normalized Difference Vegetation Index (NDVI). NDVI can be used to determine if a target object is green and healthy vegetation or not. **(C)** Aerial orthomosaic of a maize field acquired with an UAV. **(D)** Aerial orthomosaic of a maize field acquired with a UAV, in which individual plots have been segmented for trait extraction using machine learning methods to evaluate crop growth and development. **(E)** Raw image of a wheat spike infected with Fusarium head blight taken by a terrestrial phenotyping rover. **(F)** Processed image of a wheat spike infected with Fusarium head blight, with separate spike (purple mask) and pathogen (red mask) segmentation performed using a deep-learning based image analysis pipeline.

Stress-induced changes in plants can be measured at the plant, plot, or field level, with each scale offering unique insights into plant responses (Kennelly et al., 2012) (**Figure 2**). For small-scale studies, where stress traits are easy to identify, traditional methods such as visual inspection or manual measurements may suffice. However, for large-scale studies that involve numerous plants or large areas, efficient stress detection methods become crucial. In these cases, traditional phenotyping methods, such as non-automated field scouting, are time-consuming, costly, and often hindered by accuracy bottlenecks (Furbank and Tester, 2011). Remote sensing offers a solution for detecting plant stress by measuring electromagnetic signals emitted or reflected from plant surfaces (Baret et al., 2007). The technologies allow for monitoring stress occurrence and plant responses at atmospheric and aerial scales, though their resolution depends on the platform used (Sweet et al., 2022).

Satellite-based imaging platforms capture data at field or multi-field resolutions, making them useful for scouting stress presence or absence across large areas (**Figure 2A**). Since the 1980s, atmospheric remote sensing has been used to monitor drought and temperature stress via premature senescence (Barnett and Thompson, 1982). More recently, high-resolution satellite imagery has proven effective for detecting diseases and nutrient deficiencies and directly differentiating healthy and heat-stressed cropland with multiple cropping systems present (Raza et al., 2020; Dadsetan et al., 2021; Lai et al., 2024). However, the temporal revisit frequency of many commercial satellite constellations limits their utility for routine field scouting (Ju and Roy, 2008; Kuska et al., 2022). Additionally, publicly available satellite systems often lack the resolution necessary to distinguish individual plants within a field, limiting their application for more detailed analysis. As satellite technology advances, improvements in spatial resolution are expected to lead to more accurate crop classification and better field scouting capabilities (Brinkhoff et al., 2019; Jindo et al., 2021).

Aerial remote sensing using unoccupied aerial vehicles (UAVs) offers higher temporal and spatial resolution compared to satellite platforms, enabling on-demand, autonomous flights (Sweet et al., 2022) (**Figure 2C**). While UAVs provide improved resolution, they require more time to capture and process images. UAVs typically fall into two categories, multi-rotor or fixed winged. Multi-rotor UAVs are easier to operate, can fly closer to the targets, and require less space for takeoff and landing. Fixed-wing UAVs, on the other hand, offer better battery life, can cover larger areas, and fly at higher altitudes, but require more space for takeoff and landing (Hashemi-Beni et al., 2021). The higher spatial resolution of UAVs makes them ideal for plant, plot, and organ-level phenotyping, allowing for the detection of morphological, phenological, and yield responses to abiotic and biotic stress (Kefauver et al., 2017; Xavier et al., 2017; Sandino et al., 2018).

Despite their advantages, both atmospheric and aerial remote sensing are affected by environmental factors that can degrade image quality or prevent data collection. For satellite imagery, the distance between the sensor and the target exacerbates the atmospheric interference of signals. Clouds, smoke, water vapor, ozone, and other aerosols can obstruct data collection, but these atmospheric interferences can be accounted for during image processing and stress modeling using algorithms that consider factors like atmospheric profiles, sensor type, and elevation (Raspaud et al., 2018; Scheirer et al., 2018). Aerial platforms, being much closer to the target, experience less atmospheric interference. However, environmental conditions such as wind, precipitation, variable cloud cover, sun angle, and reflections from surfaces can still affect data collection quality.

To mitigate atmospheric noise and environmental impacts, proximal sensing can be used to gather data close to the target. For example, sensors can be mounted on ground-based vehicles, or imaging can be performed in controlled environments such as automated greenhouses (Elvanidi and Katsoulas, 2022; Kuroki et al., 2022) (**Figure 2E**). More recently, mobile phones have emerged as accessible tools for capturing images to support disease identification and severity classification (De Silva and Brown, 2023; Babatunde et al., 2024; McConachie et al., 2024). Proximal sensing offers higher spatial resolution than remote sensing, enabling detailed phenotyping at the individual plant, organ, and sub-organ levels (Gage et al., 2019; Young et al., 2019). However, proximal imaging in the field can disturb plant growth and introduce biases. As such, the choice of proximal sensing platform must consider factors such as plant height, architecture, row spacing, and field conditions (White and Conley, 2013). Additionally, the spatial resolution of imaging is inversely related to time, meaning that proximal sensing often requires longer imaging times than remote sensing, which can limit temporal resolution and increase costs per measurement (Mulla, 2013).

Both remote and proximal sensing platforms rely on high quality global positioning systems to assemble contiguous images and geotag plants or plots. Additionally, image processing and trait extraction require advanced computational resources (Sweet et al., 2022). Numerous open-source, community-developed applications are available for image processing and trait extraction in studies of abiotic and biotic stress responses (Knecht et al., 2016; Gehan et al., 2017; Walsh et al., 2024). However, for less-studied crops or novel stress-related traits, new trait pipelines may need to be developed.

As imaging technologies evolve, the integration of machine learning models is becoming more common to enhance the analysis of complex images. These models can identify patterns in data to predict and categorize phenotypes (Singh et al., 2016). Machine learning algorithms can be either supervised or unsupervised, depending on whether training data is manually classified by the user (Singh et al., 2016, Singh et al., 2018). Deep learning, a subset of machine learning, involves multiple processing layers where each layer builds upon the previous to make final predictions (Singh et al., 2018). These methods are particularly suited for remote sensing, where high-throughput phenotyping generates large datasets that may be difficult to analyze (De Silva and Brown, 2023). Due to the high memory and processing demands, image analysis may require dividing images into smaller jobs for parallel processing, optimizing resources in shared data systems (Miller et al., 2017).

### 3.2 Scope of sensors used for imaging plant stress response

A variety of sensor types are employed for atmospheric, aerial, and terrestrial imaging to assess plant stress responses. Red-green-blue (RGB) sensors capture light in the visible spectrum (400–700 nm) and are among the most widely used imaging tools due to their low cost and broad availability in research and commercial agriculture (Li et al., 2023; Rossotti, 1985). RGB imaging enables the assessment of plant morphology and color-based attributes, such as chlorosis, necrosis, and certain nutrient deficiencies (Dobbels and Lorenz, 2019). It is also valuable for detecting disease signs and symptoms (Görlich et al., 2021; Amarasingam et al., 2022). For example, deep learning models trained on RGB images have been used to accurately quantify Fusarium head blight severity on wheat spikes in field settings (Cooper et al., 2023) (**Figure 2F**).

Beyond disease detection, RGB imagining can capture key morphological traits. For instance, aerial RGB imagery can be processed into a digital elevation model for plant height estimation using overlapping image reconstructions (Tirado et al., 2020). Additionally, field orthomosaics derived from RGB images can be analyzed with machine learning models to distinguish plant pixels from non-plant background features, such as soil, to facilitate canopy cover quantification (Xavier et al., 2017) (**Figure 2D**). RGB imaging enables high-throughput assessment of plant responses to abiotic and biotic stress at high spatial and temporal scales compared to manual measurements.

Despite its utility, RGB imaging is constrained by its reliance on visible light, meaning that occlusions from overlapping plant structures can interfere with measurements. Additionally, stress responses occurring outside the visible spectrum cannot be detected with RGB sensors alone. Multispectral imaging sensors overcome these limitations by capturing discrete spectral bands beyond the visible spectrum, while hyperspectral sensors extend this capacity further by acquiring near-continuous reflectance data up to 2,500 nm (Maes and Steppe, 2019). These sensors enable the calculation of vegetation indices, detection of nutrient deficiencies, and prediction of anatomical and biochemical stress responses (Sarić et al., 2022). For instance, multispectral satellite sensors have been used to calculate normalized difference vegetation index (NDVI) across large areas, including research plots, to monitor plant health and identify stress conditions (Pinto et al., 2023) (**Figure 2B**).

Higher-resolution multispectral and hyperspectral platforms facilitate detailed stress phenotyping at the plant level. These sensors have been used to assess biomass accumulation, osmotic potential, chlorophyll content, and the concentration of key nutrients such as nitrogen, potassium, phosphorus, and sulfur (Mahajan et al., 2014; Severtson et al., 2016; Cotrozzi and Couture, 2020). Importantly, hyperspectral and multispectral sensors allow for early detection of plant stressors, including drought and disease, before symptoms become visible (Behmann et al., 2014). Given that even brief stress exposure can impact crop yield and nutritional quality, early detection is critical for mitigating losses (Pérez-López et al., 2015). While these sensors can identify stress-associated spectral signatures, they often require additional analytical techniques to determine causal mechanisms (Yan et al., 2014). Moreover, hyperspectral sensors can exhibit high signal-to-noise ratios, which may impact *in situ* data quality (Adão et al., 2017).

Thermal imaging is another key tool in plant stress phenotyping. Thermal sensors are cost-effective, portable, and high-resolution, allowing rapid deployment in dynamic environments (Galieni et al., 2020). These sensors measure plant surface temperature and gas exchange, making them useful for monitoring stomatal activity and detecting water stress (Pineda et al., 2020; Pradawet et al., 2023; Rippa et al., 2023). Thermal imaging has also been applied to disease and nutrient deficiency diagnosis (Kefauver et al., 2017; Matese et al., 2018). For example, thermal sensors detected wheat leaf rust up to seven days before visible symptoms emerged (Zhu et al., 2018).

In addition to spectral imaging, non-spectral sensors such as light detection and ranging (LiDAR) are used to measure plant morphological traits. LiDAR sensors emit rapid pulses of light and calculate return time to construct high-resolution 3D models of plant structures (Hosoi et al., 2011). Unlike spectral sensors, LiDAR is not affected by atmospheric conditions such as sun angle or cloud cover, making it a robust tool for assessing plant height, canopy structure, and density. LiDAR data has been used to monitor plant growth rates, detect weed competition, and assess nitrogen deficiency (Andújar et al., 2013; Eitel et al., 2014). Beyond agriculture, airborne LiDAR has also been used to predict how understory microclimate buffering can impact heat stress in forests (Gril et al., 2023). However, high costs and logistical challenges associated with LiDAR deployment currently limit its widespread use in field settings (Lohani and Ghosh, 2017).

As imaging technologies continue to advance, integrating multiple sensor types, such as RGB, multispectral, hyperspectral, thermal, and LiDAR, into plant phenotyping platforms will enhance stress detection and monitoring capabilities. Combining these sensors with machine learning approaches can further improve trait extraction and stress prediction, enabling more precise, high-throughput assessments of plant health.

### 3.3 Limitations to remote sensing for stress research

Remote sensing offers valuable tools for large-scale phenotyping of plant responses to biotic and abiotic stress, enabling early detection of stress symptoms and supporting yield and quality loss mitigation. When combined with machine learning and computer vision, remote sensing can objectively quantify plant responses and generate large, reusable datasets for ongoing research. However, several limitations must be considered when applying remote sensing technologies in stress research.

One primary limitation is the trade-off between spatial resolution and image acquisition time. Higher biological resolution typically requires increased imaging time, which can reduce the feasibility of high-throughput studies (Mulla, 2013). Additionally, remote sensing methods often provide qualitative estimates of stress responses rather than direct quantifications of stress-related factors such as nutrient concentrations or pathogen loads, which are better assessed through microscopic or biochemical techniques (Galieni et al., 2020; Nouman et al., 2022).

Another significant constraint of remote sensing is its limited applicability for studying subsoil and root conditions. Both spectral and non-spectral remote sensing technologies rely on direct line-of-sight between the sensor and the plant tissue being imaged. Instead, root system architecture is commonly assessed through shovelomics, a manual excavation technique that enables visual or computational trait measurement (Trachsel et al., 2011). While labor-intensive and requiring large field study areas, shovelomics has been successfully applied to evaluate root architectural responses to nutrient deficiencies and soil-based stress conditions (Trachsel et al., 2013).

To improve throughput in root studies, root pulling force has emerged as an alternative to traditional shovelomics. Recent studies have identified shared genetic associations between root pulling force, nutrient transport, and other root system architecture traits, highlighting its potential for large-scale root phenotyping in stress-response research (Woods et al., 2022). Beyond field-based methods, laboratory and greenhouse approaches are commonly used to supplement root studies (Walter et al., 2015). X-ray computed tomography (CT) and magnetic resonance imaging (MRI) allow for non-destructive, repeated measurements of root systems, providing detailed insights into 3D root structure and growth under controlled stress conditions (van Dusschoten et al., 2016; Kehoe et al., 2022). Advances in digital imaging have further enabled construction of 3D root models using commercially available cameras, expanding opportunities for root system analysis without specialized scanning equipment (Liu et al., 2021).

Despite technological advances in imaging platforms, sensor resolution, and data acquisition, significant challenges remain in the analysis and interpretation of remote sensing data. Machine learning and deep learning algorithms have greatly expanded the capacity to extract meaningful information from complex image datasets, but these methods often require large, well-annotated training sets to perform accurately—something not always feasible in plant stress research. To address this, data augmentation techniques such as cubic power scaling, random rotation and scaling, and parallel channel spatial attention have been used to artificially increase training datasets and highlight stress-relevant features (Bao et al., 2024). These approaches improve model learning without requiring additional annotated samples.

However, simpler methods can sometimes yield comparable results. For instance, image-based spectral indexing has been shown to differentiate healthy and diseased tissue with similar accuracy to more complex deep learning pipelines (McConachie et al., 2024). This suggests that researchers should carefully weigh the benefits of advanced machine learning models against the time, expertise, and computational resources required to develop, train, and validate them. Going forward, integrating remote sensing with complementary techniques, such as high-resolution root imaging, field-based phenotyping, and thoughtful data analyses, may help bridge current gaps in whole-plant stress phenotyping, offering more holistic insights into plant responses under field conditions.

## 4 Integrative research is required for continued advances on emerging plant stress conditions and reactions

Climate change is increasing extreme weather events, plant disease outbreaks, and soil degradation, threatening global food security (Arias et al., 2021; Rillig et al., 2021; Amirkhani et al., 2022). While molecular and omics methods characterize stress response mechanisms, remote sensing enables high-throughput monitoring at numerous scales. Integrating these approaches is essential for accurate detection, diagnosing, and understanding plant stress responses. Expanding research on multiple stress interactions, incorporating emerging technologies, and enhancing computational resources will improve stress detection and management. Strengthening data sharing and machine learning applications will further refine predictive models, ultimately supporting more resilient agricultural systems.

A key limitation in this progress, however, is the difficulty of translating laboratory findings into field applications. Many omics techniques, such as transcriptomics, metabolomics, and proteomics, rely on controlled conditions and destructive sampling, which limits their feasibility in real-world agricultural settings. Recent advances in portable platforms, including mobile qPCR devices or field-deployable biosensors, offer promising solutions for enabling *in situ* molecular diagnostics (Doi et al., 2021; Bharti et al., 2024). To enhance field applicability, experimental designs should mirror realistic environmental conditions, and interdisciplinary collaborations, particularly between researchers, breeders, agronomists, and growers, are essential to ensure that diagnostic tools are both accurate and practical. Integrating remote sensing with targeted omics-based sampling may provide a scalable framework for bridging lab and field, enabling more precise, real-time stress detection across biological and environmental gradients.

### 4.1 Multi-omic research improves stress research outcomes

Remote sensing, molecular, and omic techniques offer unique advantages for identifying plant stress and understanding host responses. However, single detection methods are often insufficient to capture the complexity of stress response mechanisms. For example, in the past three years alone, researchers have employed a wide range of experimental approaches to study heat stress across various species and natural systems (**Supplementary Table 1**). Multi-omic analysis integrates phenomic, genomic, transcriptomic, ionomic, metabolic, and other data sources, providing deeper insights than any single approach alone.

One common application combines high-throughput remotely sensed phenomic data with genomic variation to link stress response traits to genetic regions of interest. This strategy, often used in breeding and genetics, helps identify stress-resistant germplasm but lacks the resolution to pinpoint underlying biological mechanisms (Xiao et al., 2022). Expanding multi-omic integration can bridge this gap.

For example, combining phenomic, ionomic, genomic, and transcriptomic data, has distinguished salinity-resistant from susceptible rapeseed varieties while identifying key genes linked to tolerance (Zhou et al., 2021). Similarly, transcriptomic, metabolomic, and proteomic data have uncovered transcription factors and pathways involved in plant-pathogen interactions and long-term heat stress (Tang et al., 2022; Zhang et al., 2025). Metabolomic selection has been explored to predict consumer flavor perception in tomato and blueberry, with potential applications for identifying genomic regions that stabilize fruit quality under stress (Colantonio et al., 2022). A new spatial multimodal analysis protocol integrating spatial transcriptomics and MS imaging can now reveal gene expression and signaling molecule patterns in stressed versus healthy plant tissue (Yu et al., 2022). These multi-omic approaches will continue to improve our understanding of plant stress biology, uncover shared response mechanisms across different stressors, and guide future crop improvement efforts.

Despite the promise of integrating data across biological scales, significant challenges remain. Correlations between omic-level measurements (e.g. transcriptomic or metabolomic data) and field-based phenotypic traits are often weak, due to differences in spatial and temporal resolution, environmental variability, and genotype-by-environment interactions (Deery and Jones, 2021). These inconsistencies can hinder data integration and limit the predictive accuracy of machine learning models, which may overfit when trained on noisy or weakly correlated variables (Dorrepaal et al., 2016). To improve biological inference and ensure model reliability, multi-omic studies must prioritize robust ground-truthing, thoughtful experimental design, and the inclusion of independent validation datasets. Developing integrative frameworks that account for biological complexity while maintaining analytical rigor will be key to advancing the utility of multi-omics in stress phenotyping.

### 4.2 Multi-stress research reveals antagonistic interactions between concurrent abiotic and biotic stressors

Climate change is expected to increase the frequency and severity of concurrent stressors in the coming decades. For example, simultaneous heat waves and drought can intensify evapotranspiration, lead to soil desiccation and amplifying stress conditions (Fischer et al., 2007). The combined impact of multiple abiotic stressors is often more harmful than individual stress conditions. In Arabidopsis, exposure to combinations of heat, salt, excess light, acidity, heavy metals, and oxidative stress resulted in minimal effects when applied individually but became increasingly lethal as stressors accumulated. Each combination also triggered distinct gene expression profiles, demonstrating the plasticity of plant responses to complex abiotic stress conditions (Zandalinas et al., 2021).

Abiotic stress can also influence the incidence and severity of biotic stress. For instance, high humidity caused by heat waves and flooding increased fungal pathogen outbreaks (Romero et al., 2022). Similarly, heat stress and tomato yellow leaf curl virus infection in tomatoes not only exacerbated disease symptoms but also suppressed heat shock-induced protein production in later infection stages (Anfoka et al., 2016). Conversely, some stress interactions can be synergistic, enhancing plant resistance or acclimation. Short-term cold exposure in Arabidopsis primes the salicylic acid pathway, improving resistance to *Pseudomonas syringae* and potentially other hemi-biotrophic pathogens (Wu et al., 2019). These examples highlight the dual nature of stress interactions, where the outcome can be either deleterious or beneficial depending on the context.

Multifactorial stress interactions further complicate plant responses, as they can act synergistically or antagonistically depending on timing, intensity, and environmental conditions. For example, elevated atmospheric CO_2_ levels, expected to become more common due to climate change, have been shown to reduce susceptibility to foliar pathogens such as leaf rust in durum wheat. This effect is linked to stomatal closure and carbohydrate accumulation, which can trigger defense responses. However, rising temperatures associated with climate change may counteract this benefit by promoting stomatal opening and facilitating pathogen entry (Porras et al., 2023). These contrasting outcomes underscore the need for multifactorial stress studies, as single-stress analyses are increasingly insufficient for understanding the full spectrum of plant stress responses. Future research must prioritize identifying both antagonistic and synergistic interactions under combined stress scenarios to support the development of crops with robust, climate-resilient stress tolerance.

In addition to well-studied stressors like drought and heat, emerging environmental challenges such as waterlogging and microplastic contamination are drawing increased attention. Waterlogging limits oxygen availability in the rhizosphere, which impairs root respiration, disrupts nutrient uptake, and alters hormonal signaling pathways (Shabbir et al., 2022; Verslues et al., 2023). Stress detection methods for waterlogged conditions include root imaging, redox potential sensors, and monitoring chlorophyll fluorescence which reflects damage to photosynthetic function (Huang et al., 2024; Zhang et al., 2024). Meanwhile, microplastics, now frequently detected in agricultural soils, have been shown to alter root morphology, impair nutrient transport, and trigger oxidative stress responses. Detection typically involves Fourier-transform infrared (FTIR) spectroscopy and advanced imaging techniques such as electron microscopy (Sheeba et al., 2025). As climate change and anthropogenic inputs continue to reshape agroecosystems, broadening research into these emerging stressors will be essential for developing comprehensive detection tools and sustainable crop management strategies.

### 4.3 New technology expands stress detection and response research

Emerging technologies are enhancing multi-omic and multi-stress research by addressing limitations in current methods. While remote sensing is well-established for stress detection, factors such as satellite revisit frequency, resolution, and UAV payload constraints limit data acquisition. The launch of the SkySat satellite constellation in 2020 improved these capabilities, providing daily global imaging at 0.5 m resolution across four spectral bands, including infrared, to support crop monitoring and stress detection at the plot scale (Planet, 2022; Pinto et al., 2023).

Beyond remote sensing, the Agricultural Internet of Things (IoT) integrates networks of sensors to continuously monitor environmental conditions, plant growth, and disease outbreaks, supplementing existing imaging methods (Xu et al., 2022; Amogi et al., 2024). Advances in medical technology are also being repurposed for plant stress research. CRISPR-Cas12-based lateral flow assays, originally developed for rapid pathogen detection in humans, offer a promising tool for plant diagnostics with minimal lab requirements (Broughton et al., 2020). Similarly, magnetic resonance imaging (MRI), widely used in medicine, provides a non-invasive method to assess plant morphology, monitor water stress responses, and detect pathogens (Blümler et al., 2009; Hillnhütter et al., 2012; Sorin et al., 2018; Aliche et al., 2020).

New imaging techniques are further improving stress detection and response characterization. Plant Positron Emission Tomography (Plant PET) enables real-time, 3D visualization of metabolic and nutrient pathways, with applications for studying vascular transport under stress conditions (Hubeau and Steppe, 2015). Single-cell RNA sequencing offers high-resolution insights into stress responses by identifying cell types most affected and their unique gene expression profiles (Frank et al., 2022; Wang et al., 2025).

As multi-omic research expands, the resulting increase in big data requires advanced analytical approaches. Machine learning is increasingly applied to extract meaningful patterns from large datasets, including identifying key spectral features for stress detection and reducing computational complexity in hyperspectral imaging (van Dijk et al., 2021; Su et al., 2021). Cloud computing services, such as Amazon Web Services, are facilitating large-scale data processing, while improved data-sharing practices, such as open-source repositories, standardized metadata, and controlled vocabularies, will enhance collaboration and global applicability (Leonelli et al., 2017; Khan et al., 2020).

## 5 Conclusions

Abiotic and biotic stresses trigger a cascade of physiological and molecular responses in plants, many of which are not immediately visible but can significantly disrupt growth and productivity, threatening global food security. A range of omic analyses and remote sensing tools are available for stress detection and host response characterization, each with distinct advantages and limitations. As climate change intensifies extreme weather events, multi-omic and multi-stress research will be essential for identifying emerging stress conditions and resistance mechanisms. Additionally, advancements in stress detection technologies, open source data sharing, and enhanced computational resources will be critical to unifying plant stress research and mitigating climate change’s impact on food production and quality.
